# A multi-label text sentiment analysis model based on sentiment correlation modeling

**DOI:** 10.3389/fpsyg.2024.1490796

**Published:** 2024-12-20

**Authors:** Yingying Ni, Wei Ni

**Affiliations:** ^1^School of Media & Communication Shanghai Jiao Tong University, Shanghai, China; ^2^Department of Critical Care Medicine, Sir Run Run Shaw Hospital, Hangzhou, Zhejiang, China

**Keywords:** text classification, sentiment analysis, natural language processing, attention mechanism, emotion theory

## Abstract

**Objective:**

This study proposes an emotion correlation-enhanced sentiment analysis model (ECO-SAM), a sentiment correlation modeling-based multi-label sentiment analysis model.

**Methods:**

The ECO-SAM utilizes a pre-trained BERT encoder to obtain semantic embedding of input texts and then leverages a self-attention mechanism to model the semantic correlation between emotions. Additionally, it utilizes a text emotion matching neural network to make sentiment analysis for input texts.

**Results:**

The experiment results in public datasets demonstrate that compared to baseline models, the ECO-SAM obtains the precision score increasing by 13.33% at most, the recall score increasing by 3.69% at most, and the F1 score increasing by 8.44% at most. Meanwhile, the modeled sentiment semantics are interpretable.

**Limitations:**

The data modeled by the ECO-SAM are limited to text-only modality, excluding multi-modal data that could enhance classification performance. Additionally, the training data are not large-scale, and there is a lack of high-quality large-scale training data for fine-tuning sentiment analysis models.

**Conclusion:**

The ECO-SAM is capable of effectively modeling sentiment semantics and achieving excellent classification performance in many public sentiment analysis datasets.

## 1 Introduction

Sentiment analysis is a significant task in natural language processing that aims to mine the emotional tendencies of given texts, thereby helping to gain a deeper understanding of the text content and its potential impact. Currently, with the rise of public social media platforms such as Sina Weibo and Twitter, sentiment analysis techniques have shown important roles in social sentiment analysis and event tracking (Mujahid et al., 2021; Omar and Abd El-Hafeez, 2023)^.^ Relevant researchers use sentiment analysis algorithms to identify the emotional tendencies of massive social media platform users’ posts, thereby comprehensively analyzing the trend of public opinion and taking corresponding measures. The sentiment analysis technology itself has also expanded from the traditional simple binary classification task to the multi-classification task, that is, identifying the specific emotions contained in the text, such as happy, sad, like, and angry.

However, compared to the traditional binary sentiment analysis, the multi-label sentiment analysis task faces challenges such as data sparsity, class imbalance, and difficulty in modeling emotional semantics. To this end, researchers have proposed various multi-label text emotion classification models based on statistics, machine learning, and deep learning techniques. For example, sentiment analysis models based on emotional dictionaries (Wu et al., 2019; Acerbi et al., 2023; Duan et al., 2021a) identify the emotional categories of texts by matching the retrieved words in the emotional dictionary. Text emotion dictionary models based on Naive Bayes and support vector machines (Neethu and Rajasree, 2013) use statistical learning methods to analyze and model word frequency statistical features to recognize the probability of text emotions. With the widespread application of deep learning in the field of natural language understanding (Vaswani et al., 2023; Mikolov et al., 2013; Yadav and Vishwakarma, 2020; Beridge et al., 2022), deep learning text emotion recognition models represented by recurrent neural networks (RNN) (Zhang et al., 2019) and large-scale pre-trained models (pre-trained model) (Duan et al., 2021b; Cortiz, 2021) have made significant progress in the identification of specific text emotion categories by relying on the powerful capabilities of deep learning in semantic representation modeling.

To efficiently mine and utilize semantic correlation between emotions to enhance multi-label sentiment analysis, in this study, we propose an emotion correlation-enhanced sentiment analysis model (ECO-SAM). Inspired by the widely used self-attention mechanism for language modeling and the basic emotion theory, we first design a novel attention-based emotion correlation modeling module that could automatically learn the semantic correlation between emotions from data and obtain correlation-enhanced emotion embedding representation. Next, we transform the multi-label sentiment analysis problem into an information retrieval problem, which aims to find the most suitable emotions from the emotion candidate list for a given query text. Then, we design an emotion-matching module that uses neural networks to learn the matching function between emotion and text embedding from data. Finally, we demonstrate the effectiveness of ECO-SAM via extensive experiments on two public sentiment analysis datasets. The experiment results unveil that the ECO-SAM obtains the precision score increasing by 13.33% at most, the recall score increasing by 3.69% at most, and the F1 score increasing by 8.44% at most. Meanwhile, the modeled sentiment semantics are interpretable.

## 2 Related work

### 2.1 Basic emotion theory

The basic emotion theory was proposed by American psychologist Ekman (1972). The theory believes that humans have six basic emotions: happiness, sadness, fear, anger, surprise, and disgust. These basic emotions are considered to be universally present across cultures and species. Based on the basic emotion theory, Ekman (1972) found some universality of emotional expressions through observing the facial expressions of people in different cultures. Izard (1977) expanded the basic emotion theory, discussing the relationship between basic emotions and the relationship between emotion and cognition. The study proposed a model of the emotional system, describing the relationships between basic emotions and how they interact and regulate each other. For example, the author pointed out that there is a close relationship between “anger” and “disgust,” while “happiness” and “sadness” have an antagonistic relationship. Russell (1980) proposed the circular emotion theory, which expanded the basic emotion theory and emphasized the construction and subjective experience of emotions, implying the idea of modeling the association between emotions. Cowen and Keltner (2017) explored how people describe and distinguish different emotional experiences in self-reports. The study found more fine-grained emotional experiences compared to the basic emotion theory, expanding the understanding of emotions and breaking through the traditional concept of basic emotions. It shows that emotions are complex and diverse and can be described and captured through multiple discrete emotion categories and continuous gradients.

In summary, the basic emotion theory first proposed the six basic elements of emotion. Relevant scholars have delved deeper into the construction of emotions and the relationships between emotions based on the basic emotion theory and developed a gradually more comprehensive emotional theory framework.

### 2.2 Sentiment analysis

Sentiment analysis is a text classification task that aims to identify the emotional category of a text based on its semantic features. According to the different distribution of emotion labels, sentiment analysis can be divided into emotion polarity classification (binary), emotion category classification (multi-class), and emotion label classification (multi-label). Sentiment analysis models include rule-based emotion dictionary methods (Wu et al., 2019; Acerbi et al., 2023; Xu et al., 2021), statistical machine learning-based methods (Neethu and Rajasree, 2013; Rastogi et al., 2021), and deep learning-based methods (Yadav and Vishwakarma, 2020; Duan et al., 2021a; Cortiz, 2021; Zhang et al., 2019; Ahmed et al., 2022). The rule-based emotion dictionary method is an unsupervised approach that uses emotion dictionaries to obtain the emotion values of emotional words in the document and then determines the overall emotional tendency of the document through weighted calculation. This method does not consider the connections between words, nor does it consider the changes in the emotional tendency of words due to the context.

Common emotion dictionaries include English dictionaries such as General Inquirer, SentiWordNet, Opinion Lexicon, and MPQA (Chatterjee et al., 2019), as well as Chinese dictionaries such as HowNet (Fu et al., 2017), NTUSD (Chen et al., 2022), and the Chinese emotion lexicon ontology (Deng et al., 2017). The statistical machine learning-based method is a supervised approach that trains machine learning classification models on text data with emotion labels and then applies the trained machine learning classification models to text emotion prediction tasks. For example, Gaye et al. (2021) proposed a text emotion recognition model based on support vector machines (SVMs), dividing the emotion analysis process into two strategies and four methods. Ghourabi et al. (2020) proposed a text emotion recognition method based on Naive Bayes, establishing a three-layer tree-structured emotion recognition structure. In addition, Patel and Urry (2024) proposed a text emotion recognition method that combines deep semantic and surface-level grammar, applicable to aspect-level sentiment analysis.

The deep learning-based method is also a supervised approach, training neural network classification models on text data with emotion labels, and utilizing the strong fitting ability of neural networks to accurately predict text emotion categories. For example, Grandjean et al. (2008) proposed a sentiment analysis model based on convolutional neural networks, where the dual convolutional layer structure can extract features from sentences of any length. Ji et al. (2019) proposed a sentiment analysis model based on deep belief networks, solving the problem of sparse text features. With the rise of large language models (LLMs) (Zhao et al., 2023; Elyoseph et al., 2023; Liu et al., 2023), the pre-trained LLM-based methods have emerged in sentiment analysis and achieved excellent performance on large-scale datasets. For instance, Valderrama et al. (2024) used the BERT model to obtain more complete text semantic representations, thereby more accurately predicting text emotion categories. Sailunaz et al. (2018) compared the sentiment analysis capabilities of various large language models in the research on user behaviors of spreading others’ privacy information on social networks. Gao et al. (2021) proposed to use prompt learning to enhance the classification performance of pre-trained models when the data volume is relatively small. In the multi-modal emotion recognition scenario, Zhu et al. (2020) proposed a sentiment analysis model based on improved ResNet to analyze and improve the accuracy of image emotion classification. Currently, deep learning models play a pivotal role in accurate sentiment analysis. As shown in Table 1, we count and list current state-of-the-art sentiment analysis methods based on previous research.

**Table 1:** Current state-of-the-art (SOTA) sentiment analysis methods.

Reference	Dataset	Task type	SOTA model	Score
Sachin et al. (2020)	Amazon review	Binary	LSTM	Acc = 0.70
Demszky et al. (2020)	GoEmotions-all	Multi-label	BERT	Macro F1 = 0.64
Brauwers and Frasincar (2022)	Online phone review	Binary	LSTM + LDA	Acc = 89.5
Abdullah and Ahmet (2022)	Sentihood	Binary	BERT	Acc = 0.94
Bashiri and Naderi (2024)	Narr-KDML-2012	Binary	T5	F1 = 0.94

### 2.3 Deep learning and attention mechanism

The attention mechanism was first proposed by Bahdanau et al. (2016), which is a deep learning technique used to model the semantic association and related representation between different parts of the semantic sequence. In natural language processing, the attention mechanism is often used to model the semantic association between the context in the corpus, thereby achieving the correspondence between the model output results and the context in tasks such as text generation and text classification. The transformer model proposed by Vaswani et al. (2023) is a representative model using a self-attention mechanism. The transformer model has strong semantic representation and text output capabilities and is the foundation of many text classifiers and text sentiment recognition methods.

## 3 Sentiment analysis based on emotion correlation modeling

Existing sentiment analysis methods struggle to model the important role of emotion correlation in emotion recognition. Therefore, this study first proposes a text sentiment analysis method based on emotion correlation modeling (ECO-SAM). Subsequently, the superiority of the ECO-SAM in sentiment analysis and emotion correlation modeling is demonstrated on the Weibo text sentiment analysis dataset. Finally, the ECO-SAM is applied to text emotion analysis under a given topic.

### 3.1 An overview of ECO-SAM

**Figure 1:**
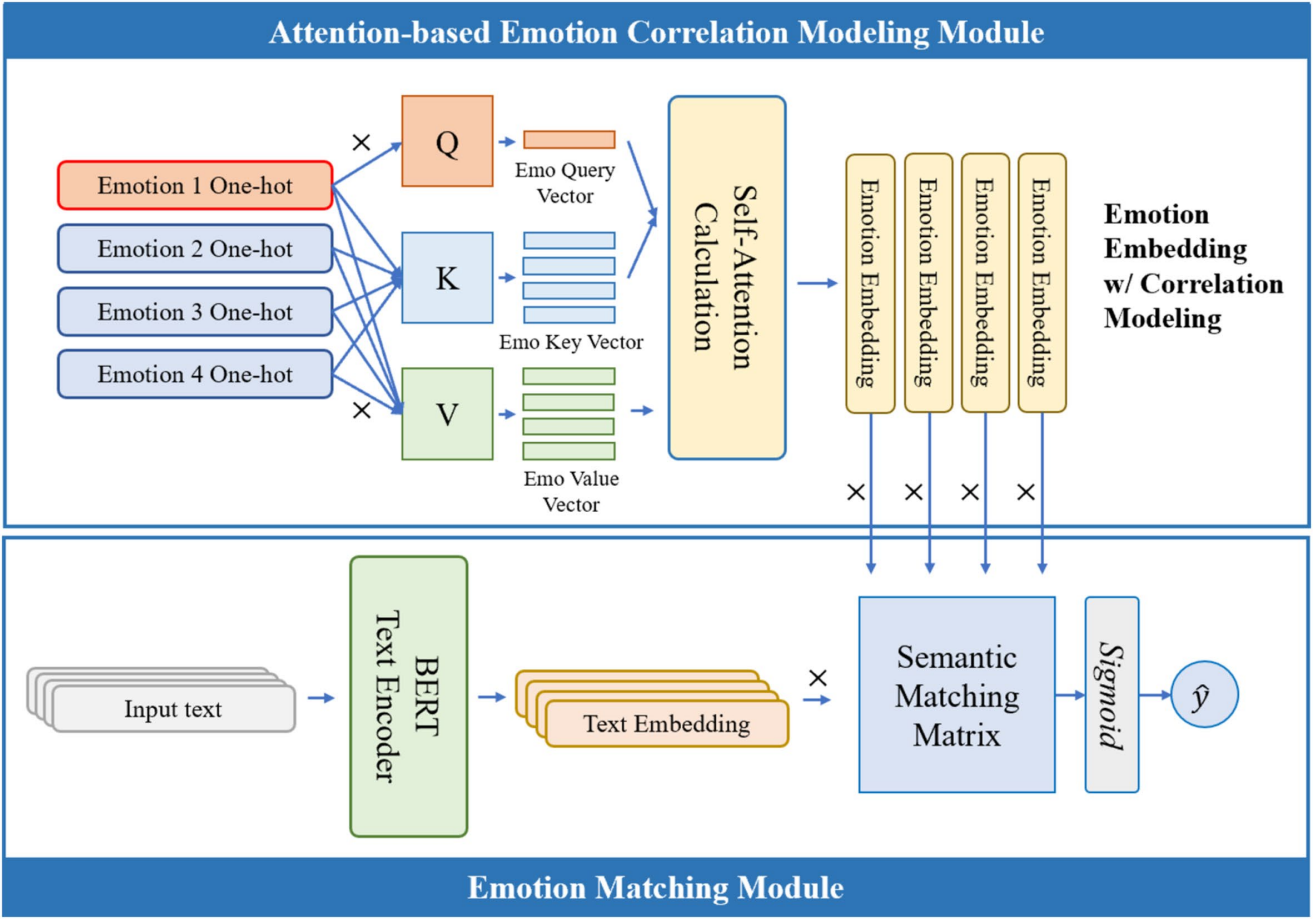
Structure of ECO-SAM.

The framework of the proposed ECO-SAM algorithm is shown in Figure 1. The framework consists of three modules: the text encoder module, the attention text correlation modeling module, and the emotion matching module. The text encoder module uses the large-scale pre-trained model BERT to encode the text input into a high-dimensional text semantic vector. The attention text correlation modeling module uses the attention mechanism to transform the trainable emotion inherent feature vectors and output feature vectors containing emotion correlations, while also outputting an emotion correlation matrix. The emotion classification neural network module matches the text semantic vector with each emotion-correlated emotion feature vector and calculates the probability of the text containing that emotion. The algorithm finally outputs the probability of emotion containment.

In the training stage, the model’s emotion inherent feature vectors, attention text correlation modeling module, and emotion classification neural network are trained using a multi-label text emotion recognition dataset. In the inference stage, the parameters of ECO-SAM are frozen to achieve end-to-end sentiment analysis.

### 3.2 BERT text encoder

The BERT text encoder in the ECO-SAM (Cui et al., 2021) is a large-scale pre-trained text encoding model based on BERT (Vaswani et al., 2023). This module utilizes a masked language model (MLM) to generate deep bidirectional language representations. Experiments in the original BERT study (Vaswani et al., 2023) have demonstrated that BERT achieved state-of-the-art performance on 11 natural language processing tasks, which substantiates the efficacy of the BERT module in text semantic representation.

Formally, let the original text input be a character sequence $s=\left\{w_{1},w_{2},\ldots ,w_{N}\right\}$ , then the encoding process of BERT can be formalized as shown in Equation 1:


(1) $$v_{s}^{\left(\mathrm{senti}\right)}=f_{\mathrm{BERT}}\left(w_{1},\ldots ,w_{N}\right)$$

where $v_{s}^{\left(\mathrm{senti}\right)}\in {\mathbb{R}}^{D}$ , is the text semantic representation vector. The D represents the dimension of the text semantic representation vector defined by BERT. In general, D =1,024 .

### 3.3 Attention-based emotion correlation modeling module

The attention-based emotion correlation modeling module uses the self-attention mechanism to model the semantic correlation of emotions, thereby addressing the lack of research on emotion correlation in existing studies. Specifically, the self-attention mechanism adopts the query-key-value (QKV) pattern. Each emotion in the framework has a trainable query vector, key vector, and value vector (the value vector corresponds to the inherent emotion feature vector in Figure 1). First, for a target emotion, its query vector is obtained, and the cosine similarity between the query vector and the key vector of each other emotion is calculated. The similarity with each other emotion reflects the semantic dependence of the target emotion, i.e., the extent to which the semantic representation of the target emotion depends on that particular emotion. Then, the feature vector containing the emotion correlation of the target emotion is calculated. This vector is the weighted average of the inherent feature vectors (value vectors) of each emotion, with the weights being the calculated semantic dependence. Finally, Pearson’s correlation coefficient between the feature vectors containing emotion correlations is calculated and the emotion correlation matrix is output.

Formally, one-hot encoding is used to mark each emotion. Let $S={\left(s_{jk}\right)}_{D\times K}$ denotes the emotion feature inherent vector matrix, $Q={\left(q_{jk}\right)}_{D\times K}$ denotes the evaluation query vector matrix, and $Z={\left(z_{jk}\right)}_{D\times K}$ denotes the emotion key vector matrix. In the prediction of emotion probabilities, this module first calculates the emotion feature $e_{k}$ using S and the one-hot emotion vector $x_{k}$ , as shown in Equation 2. Meanwhile, it obtains the query and key vectors for each emotion, as shown in Equation 3 and Equation 4:


(2) $$e_{k}=S\times x_{k}\text{,}$$

(3) $$q_{j}=Q\times x_{j},j=1,2,\ldots ,K\text{,}$$

(4) $$z_{j}=Z\times x_{j},j=1,2,\ldots ,K\text{,}$$

Subsequently, the semantic dependence similarity between the target emotion and each emotion is calculated using Equation 5:


(5) $$\alpha_{k,j}=\mathrm{softmax}\left(\frac{q_{k}^{⊤}z_{j}}{\sqrt{D}}\right)\text{.}$$

Finally, the emotion-semantic embedding with correlation modeling for the target emotion is calculated, as presented in Equation 6:


(6) $$e_{k}^{\left(att\right)}=\overset{K}{\underset{j=1}{\sum }}\alpha_{k,j}\times e_{j}\text{.}$$

The resulting calculation $e_{k}^{\left(att\right)}$ is the emotion vector representation that contains the emotion dependence relationship, which is used in the subsequent steps to recognize the emotion of the text.

### 3.4 Emotion matching module

The emotion matching module uses a neural network to compute the degree of matching between the text semantic representation and the emotion-semantic representation, thereby predicting the probability of each emotion in the text. Specifically, given the semantic representation vector of a sentence and the semantic representation vector of an emotion, this module uses a quadratic form neural network to predict the probability of the text emotion, as shown in Equation 7:


(7) $$\begin{array}{c} {\hat{y}}_{s,k}=\mathrm{sigmoid}\left(v_{s}^{\left(att\right)⊤}We_{k}^{\left(\mathrm{senti}\right)}\right)\;\\ =\mathrm{sigmoid}\left(v_{s}^{\left(att\right)⊤}\left(O^{⊤}\Lambda O\right)e_{k}^{\left(\mathrm{senti}\right)}\right)\\ =\mathrm{sigmoid}\left({\left(\;Ov_{s}^{\left(att\right)}\right)}^{⊤}\Lambda \left(Oe_{k}^{\left(\mathrm{senti}\right)}\right)\right) \end{array}$$

where $W=O^{⊤}\Lambda O$ is the eigenvalue decomposition of the semantic matching matrix $W\in {\mathbb{R}}^{D\times D}$ . The above eigenvalue decomposition transformation implies that this neural network prediction process is equivalent to applying the same linear transformation to the text semantic vector and the emotion-semantic vector and then taking the element-wise weighted average, with the weights being the eigenvectors. The training process of the neural network is equivalent to optimizing the linear transformation and the eigenvectors, so that the predicted probability of text emotion is close to the true data label.

### 3.5 Loss function

Since the sentiment analysis problem addressed by the ECO-SAM is a multi-label classification problem, the cross-entropy loss is used as the loss function, as shown in Equation 8. During the model training process, the training objective of the ECO-SAM is to minimize the loss function value:


(8) $$L\left(\Omega \right)=-\overset{N}{\underset{i=1}{\sum }}\overset{C}{\underset{k=1}{\sum }}y_{i,k}\log\left(p_{i,k}\right)$$

where Ω represents all the trainable parameters in the ECO-SAM. N represents the number of samples (text samples in the training set). C represents the number of possible emotion categories. $y_{i,k}$ represents whether the text contains the emotion, where $y_{i,k}=1$ indicates the text contains the emotion k , and $y_{i,k}=0$ indicates otherwise. $p_{i,k}$ represents the probability predicted by the ECO-SAM that the text contains each emotion.

## 4 Text emotion recognition experiment

### 4.1 Experimental setup

This experiment compares the proposed multi-label sentiment analysis model, ECO-SAM, with various baseline text emotion prediction models using the public Weibo dataset. The goal is to verify the accuracy of the ECO-SAM in sentiment analysis and its ability to model emotion feature correlations. For the experimental datasets, this study used two publicly available datasets: NLPCC2014 and GoEmotions (Demszky et al., 2020). This module takes three inputs for emotion k: the feature inherent vector, query vector, and the corresponding key vectors for all emotions. Each text contains up to two emotions. The GoEmotions dataset consists of 58,000 text data from the English forum Reddit, with the original data containing 27 fine-grained emotion categories. Based on the basic emotion theory, we screened out the 7 emotions consistent with the NLPCC2014 dataset as well as the neutral case as the target of sentiment analysis and selected 32,445 valid samples. Next, we split each dataset into training, validation, and test sets in the ratio of 70%:10%:20%, respectively.

In terms of the experiment setting, all models implemented using Python 3.8, with the deep learning framework being PyTorch, and the operating system being Linux. The hardware configuration for running the experiments is a server with two 2.10GHz Intel Xeon E5-2620 v4 CPUs and one NVIDIA Tesla-A100 GPU.

### 4.2 Text emotion prediction experiment

The main experiments in this study include emotion prediction experiments and emotion feature correlation analysis. Finally, the ECO-SAM emotion prediction model is applied to sentiment analysis. In the emotion prediction experiment, the following baseline models are used:

Random: Random prediction. For each emotion, the text has a 1/2 probability of being classified into that emotion category. Whether an emotion prediction model performs better than random prediction is a basic criterion for its usability.

cnsenti (Deng and Nan, 2022): Chinese Sentiment, an emotion prediction model based on the HowNet emotion dictionary of Chinese Knowledge Network.

SVM (Mullen and Collier, 2004): Support Vector Machine, an emotion prediction model based on support vectors. In the experiment, BERT is used to encode the text into semantic vectors, which are then used as input to the SVM.

LSTM (Hochreiter and Schmidhuber, 1997): Long short-term memory is a type of recurrent neural network (RNN) architecture designed to address the vanishing gradient problem in traditional RNNs. LSTMs are particularly effective at learning long-term dependencies in data, making them well-suited for applications such as sentiment analysis and time series analysis.

BiLSTM (Graves and Schmidhuber, 2005): It is an extension of the traditional LSTM architecture that processes input sequences in both forward and backward directions. This bidirectional approach provides a more comprehensive understanding of the sequence.

BERT (Devlin et al., 2019) is a pre-trained transformer-based language model. BERT can encode raw texts into semantic vectors with rich information for downstream tasks. For the text emotion prediction task, we use a fully connected neural network as the downstream output layer.

T5 (Raffel et al., 2020): It is a transformer-based language model proposed by Google that unifies various NLP tasks by framing them all as text-to-text problems, where both input and output are text strings.

The results of the text emotion prediction experiment are shown in Table 2. Considering the characteristics of the multi-label classification task, the evaluation metrics are Micro Precision, Micro Recall, and Micro F1 Score. The higher the score for each of these evaluation metrics, the higher the accuracy of the model’s text emotion prediction.

**Table 2:** Statistics of datasets after preprocessing.

Statistics	NLPCC2014	GoEmotions
# Samples	45,421	32,445
Ratio of samples without emotion	44.1%	56.3%
Ratio of samples w/ 1 emotion	38.4%	41.4%
Ratio of samples w/ 2 emotions	17.5%	2.2%
Ratio of samples w/ 3 emotions	0.0%	0.06%
Ratio of samples w/ 4 emotions	0.0%	0.01%

Since GoEmotions is an English dataset, the baseline model cnsenti, which is based on the Chinese dictionary, is unable to recognize the text emotions in this dataset. From the above experimental results, it can be seen that the ECO-SAM proposed in this study outperforms the existing text emotion prediction baseline models in terms of precision, recall, and F1 score, with the highest increase in precision being 13.33%, the highest increase in recall being 3.69%, and the highest increase in F1 score being 8.44%. This proves that the ECO-SAM can predict text emotions more accurately compared to existing models (Table 3).

**Table 3:** Experiment results of sentiment analysis.

Dataset	NLPCC2014			GoEmotions		
Model	Precision ↑	Recall ↑	F1 score ↑	Precision ↑	Recall ↑	F1 score ↑
Random	0.1989	0.4929	0.2636	0.1260	0.4894	0.2005
cnsenti	0.1403	0.0943	0.1084	-	-	-
SVM	0.3126	0.5005	0.3247	0.2095	0.4156	0.2786
LSTM	0.6189	0.5970	0.6077	0.5153	0.5480	0.5312
BiLSTM	0.6957	0.5843	0.6351	0.5712	0.5849	0.5779
T5	0.4150	0.4539	0.4336	0.3031	0.6338	0.4101
BERT	0.7215	0.6856	0.6740	0.5546	0.5403	0.5473
ECO-SAM	**0.8177**	**0.7029**	**0.7309**	**0.5808**	**0.6581**	**0.6170**
Improvement	**13.33%**	**2.52%**	**8.44%**	**1.68%**	**3.69%**	**6.68%**

Bold values indicate the best performance for the metric; underline values indicate the second best performance for the metric (only worse than bold values).

Furthermore, among the baseline models, the BERT method also significantly outperforms other existing methods. The comparison between the BERT and cnsenti shows that the text emotion prediction model based on BERT pre-trained language encoding has better performance on Weibo emotion prediction than the traditional model based on rules and emotion dictionaries. The comparison between BERT and SVM shows that the text emotion prediction algorithm based on neural networks has better performance on Weibo emotion prediction than the algorithm based on SVM. Compared to the best baseline model BERT, our proposed ECO-SAM method further improves the performance of the text emotion prediction model based on BERT pre-trained language encoding through an innovative emotion feature modeling module.

### 4.3 Visualization experiment: emotion feature correlation modeling experiment

**Figure 2:**
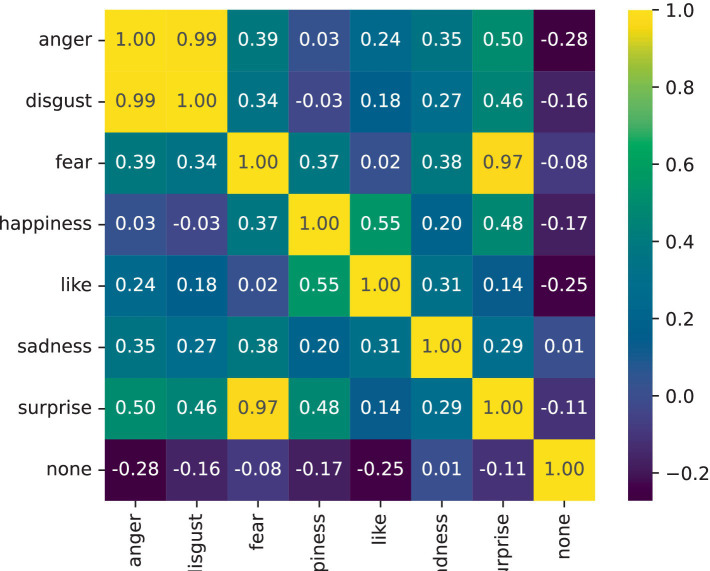
Heatmap of the correlation of emotion features.

This section uses the NLPCC2014 dataset as an example to analyze the ability of the ECO-SAM to model emotional semantic similarity. The ECO-SAM text emotion prediction model improves the accuracy of text emotion prediction by modeling the correlation between emotion features through the attention-based emotion modeling module. This experimental stage mainly focuses on the modeling results of the emotional feature correlation in the ECO-SAM. In the ECO-SAM, emotional features are represented as $e_{k}^{\left(att\right)}$ , where k represents the emotion category sequence number. For any two emotions k1 and k2, this experiment uses Pearson’s correlation coefficient of the emotion features as the measure of emotion feature correlation, denoted as $\mathrm{Corr}\left(k_{1},k_{2}\right)$ . This correlation coefficient ranges between −1 and 1. When $\mathrm{Corr}\left(k_{1},k_{2}\right)>0$ , the two emotion features are positively correlated (similar); when $\mathrm{Corr}\left(k_{1},k_{2}\right)\approx 0$ , the two emotion features are uncorrelated (independent); when $\mathrm{Corr}\left(k_{1},k_{2}\right)<0$ , the two emotion features are negatively correlated (semantically opposite). The results of the emotion feature correlation calculation are shown in the following figure, which includes seven emotions: anger, disgust, fear, happiness, like, sadness, and surprise. The brighter the color of each square in the figure, the greater the correlation value, and the stronger the association between the two emotions. According to Figure 2, the three emotions most strongly associated with each emotion are as follows:

- Anger: Disgust (0.99), Surprise (0.50), Fear (0.39).
- Disgust: Anger (0.99), Surprise (0.46), Fear (0.34).
- Fear: Surprise (0.97), Anger (0.39), Sadness (0.38).
- Happiness: Like (0.55), Surprise (0.48), Fear (0.37).
- Like: Happiness (0.55), Sadness (0.31), Anger (0.24).
- Sadness: Fear (0.38), Anger (0.35), Like (0.31).
- Surprise: Fear (0.97), Anger (0.50), Happiness (0.48).

The above results show that different types of emotions, due to their semantic differences, either exhibit strong correlations or are mutually independent of each other. Some emotions, due to the consistency of their semantics, often exhibit a relatively strong clustering feature. For example, “anger” and “disgust” are both negative emotions, and their semantic correlation reaches 0.99. They also have relatively strong correlations with “fear,” indicating that the above four emotions are similar in semantic connotation, which is consistent with people’s intuition. At the same time, “happiness” and “like” have a relatively strong correlation, indicating that the two intuitively positive emotions also have similar semantic connotations. In addition, “surprise” has a relatively high semantic similarity with positive emotions such as “happiness,” as well as with negative emotions such as “fear.” This suggests that “surprise” as an emotion that an individual perceives due to sudden changes tends to be neutral. In other words, “surprise” can coexist with positive emotions (such as “pleasant surprise”) and also with negative emotions (such as “horrifying surprise”).

## 5 Discussion

The significance of this research is as follows: First, at the theoretical level, this study organically combines basic emotion theory and deep learning technology, innovatively proposes a large-scale pre-trained text emotion recognition method (ECO-SAM), and verifies the method’s accurate text emotion recognition and emotion-semantic correlation modeling capabilities through large-scale experiments on real datasets. In the task of sentiment analysis, accuracy is a core issue in related research and is also an important technical guarantee for public opinion monitoring. Therefore, the high performance of ECO-SAM in the experiments is undoubtedly of great significance for enhancing the effectiveness of public opinion monitoring. Second, by leveraging the emotion -semantic correlation modeling capability of ECO-SAM, this study also analyzes the correlation relationships between different emotions within this topic, providing important data references for related public opinion monitoring.

At the same time, this research still has some limitations. First, due to the limitations of available data, the training corpus built using the ECO-SAM is still not sufficient to fully unleash the model’s maximum performance, and the data volume needs to be further increased in future research. Second, in terms of text semantic parsing capability, the performance of the ECO-SAM method in recognizing the emotions of texts with large implicit information such as irony and sarcasm still needs to be improved. In the future research plan, on the one hand, we can further improve the text emotion recognition capability through methods such as expanding the dataset and optimizing the model architecture. On the other hand, with the rise of large language models (LLMs) (such as ChatGPT), we can combine the advantages of LLMs in text generation and emergent capabilities, as well as the advantages of ECO-SAM in strong semantic modeling and low computational cost, to develop more efficient sentiment analysis techniques. Furthermore, the topic and user distribution on online social platforms are complex and rich in information. How to leverage the rich topic and user information to assist text emotion recognition and public opinion monitoring, and explore the downstream applications of emotion recognition and emotion-semantic modeling, we also believe, is an important future research direction.

## 6 Conclusion

Online social platforms are highly susceptible to large-scale controversial network issues, many of which can easily escalate into emotionally charged irrational propagation. Existing sentiment analysis models have difficulty in modeling emotion correlation, and the accuracy of emotion prediction needs to be improved. To solve the above problems, this study first conducted extensive and in-depth-related research and innovatively proposed an emotion correlation-enhanced sentiment analysis model (ECO-SAM) based on basic emotion theory and deep learning technology, to achieve accurate text emotion recognition and emotion correlation modeling on online social platforms. The large-scale comparative experiments on the real text emotion recognition Chinese dataset NLPCC2014 and the English dataset GoEmotions verified the accurate text emotion recognition capability of the ECO-SAM. Emotion recognition comparative experiments showed that the ECO-SAM improved the precision, recall, and F1 score of text emotion recognition by 13.33, 3.69, and 8.44%, respectively, compared to the optimal baseline method BERT, effectively improving the accuracy of text emotion recognition. The emotion feature correlation experiment showed that emotions with similar emotional colors (positive/negative) have relatively strong semantic correlations; the “surprise” emotion has a relatively high semantic correlation with both positive emotions and negative emotions, acting as a bridge between the two in the emotion correlation graph.

## Data Availability

The datasets presented in this study can be found in online repositories. The names of the repository/repositories and accession number(s) can be found at: https://github.com/qweraqq/NLPCC2014_sentiment.
